# Modulation Recognition Method for Underwater Acoustic Communication Signals Based on Passive Time Reversal-Autoencoder with the Synchronous Signals

**DOI:** 10.3390/s23135997

**Published:** 2023-06-28

**Authors:** Yalin Hu, Jixin Bao, Wanzhong Sun, Xiaomei Fu

**Affiliations:** School of Marine Science and Technology, Tianjin University, Tianjin 300072, China; hyl0521@tju.edu.cn (Y.H.); bjx_wellbeing@tju.edu.cn (J.B.); fuxiaomei@tju.edu.cn (X.F.)

**Keywords:** modulation recognition, underwater acoustic communication, multipath effect, synchronization signals, passive time reversal-autoencoder, convolutional neural network

## Abstract

In the process of the modulation recognition of underwater acoustic communication signals, the multipath effect seriously interferes with the signal characteristics, reducing modulation recognition accuracy. The existing methods passively improve the accuracy from the perspective of selecting appropriate signal features, lacking specialized preprocessing for suppressing multipath effects. So, the accuracy improvement of the designed modulation recognition models is limited, and the adaptability to environmental changes is poor. The method proposed in this paper actively utilizes common synchronous signals in underwater acoustic communication as detection signals to achieve passive time reversal without external signals and designs a passive time reversal-autoencoder to suppress multipath effects, enhance signals’ features, and improve modulation recognition accuracy and environmental adaptability. Firstly, synchronous signals are identified and estimated. Subsequently, a passive time reversal-autoencoder is designed to enhance power spectrum and square spectrum features. Finally, a modulation classification is performed using a convolutional neural network. The model is trained in simulation channels generated by Bellhop and tested in actual channels which are different from the training period. The average recognition accuracy of the six modulated signals is improved by 10% compared to existing passive modulation recognition methods, indicating good environmental adaptability as well.

## 1. Introduction

Communication signal modulation recognition is based on the non-cooperative scenario between senders and receivers, which plays an important role in information recovery. In the field of wireless communication, modulation recognition is mostly based on in-phase and quadrature (IQ) samples [1,2,3], high-order Cumulant characteristics [4,5], signal instantaneous characteristics, and wavelet transforms characteristics [6], and then appropriate classifiers are designed to classify modulation types. However, these recognition methods are not suitable for complex and variable underwater acoustic channels.

With the increasing status of the ocean, more and more researchers are devoted to the modulation recognition research of underwater acoustic communication signals. Zhang et al. [7] used machine learning algorithms to recognize modulation based on cumulant, power spectral density, instantaneous phase, instantaneous phase, and frequency characteristics. Denis Stanescu et al. [8] used phase diagram entropy to characterize and identify various modulation types. Dai et al. [9] carried out wavelet denoising and time–frequency feature extraction for the received signal and used the decision tree model for modulation recognition. Huang et al. [10] extracted entropy features and morphological features, and designed optimized autoencoder (OAE) and evaluation-enhanced k-nearest neighbor (EEKNN) algorithms to recognize modulation types.

Deep learning-based methods have been continuously developed in recent years, gradually improving recognition performance. Existing methods are usually based on two types of features in classification: time-domain features and frequency-domain features.

The first type is to use time-domain features as the recognition criteria. Alex et al. [11] designed CNN for modulation recognition of received time-domain signals. Li et al. [12] designed a feature extraction and recognition network based on Resnet to classify the time-domain signals. Yao et al. [13] trained generative adversarial networks (GANs) based on time-domain waveform features for signal enhancement, feature extraction, and automatic modulation classification. Yu et al. [14] utilized long- and short-term memory (LSTM) for modulation recognition with the signals’ instantaneous characteristics. Zhang et al. [15] trained a cyclic convolutional neural network with a normalized time series. Kong et al. [16] used IQ symbols to train a residual network. Wang et al. [17] proposed a sequence convolutional network to achieve modulation classification based on signals’ temporal characteristics. Liu et al. [18] utilized principal component analysis technology to compress the original time-domain signals and then designed a deep heterogeneous network for modulation recognition. Xiao et al. [19] designed a CNN for classification based on IQ signals. However, time-domain characteristics are easily interfered with by the complex underwater acoustic channel. The above methods can only achieve ideal results when the channel conditions of the test set are the same as the training set. Once the conditions are inconsistent, the recognition accuracy will be seriously affected.

The second type is to select frequency-domain features as the recognition basis. The power spectrum, time-frequency map, frequency spectrum, and singular spectrum are common features used for modulation recognition [20,21,22,23,24]. Jiang et al. [20] proposed a sparse automatic encoder (SAE) for feature extraction and modulation recognition based on power spectrum features. Wang Bin et al. [21] used a denoising autoencoder (DAE) to denoise signals and then used CNN to classify the modulation types based on power spectral features. Wang et al. [22] proposed a relational network and fed it with power spectrums. Xu et al. [23] trained CNN with time–frequency map features of signals. Kou et al. [24] extracted the real and imaginary parts of the signal through the Fast Fourier transform (FFT) and then designed an artificial neural network (ANN) as a feature classifier.

However, the characteristics of multiple phase shift keying (MPSK) are too similar and difficult to distinguish, choosing only one feature has certain limitations. Therefore, some methods choose two features to distinguish MPSK. Jiang et al. [25] used principal component analysis to extract effective features from the power spectrums and square spectrums, distinguishing across multiple frequency shift keying (MFSK), binary phase shift keying (BPSK), and quadrature phase shift keying (QPSK). Li et al. [26] combined time-domain and frequency-domain features to classify modulation types. Firstly, MPSK and other signals were identified through time-domain waveform features, and then the square spectrum features were selected to identify BPSK and QPSK. Compared to time-domain features, frequency-domain features have a stronger anti-interference ability, but a single frequency-domain feature has limitations. This paper selects two frequency-domain features, power spectrum and square spectrum, to classify modulation types.

The severe multipath effect of underwater acoustic channels can have serious interference with the time–frequency characteristics, reducing the accuracy of modulation recognition. The existing methods passively focus on the selection of signal features, and cannot actively weaken the impact of the environment, resulting in a sharp decline in the recognition performance when underwater acoustic channel conditions change. This paper actively utilizes commonly used synchronous signals in communication as the detection signals and designs a passive time reversal-autoencoder to improve accuracy and environmental adaptability. Firstly, we identify and estimate the types and parameters of synchronization signals, and use the recovered synchronization signals as detection signals in passive time reversal. Then, we design a passive time reversal-autoencoder (PTR-AE) for multipath suppression and signal feature enhancement. Finally, modulation recognition is performed by using CNN. The modulation classification network is trained with simulation data and tested in actual underwater acoustic channel environments which are different from the training environments. We compare the proposed model to verify its effectiveness with existing methods.

## 2. Signal Model

The underwater acoustic communication signal model can be expressed as:

(1) y(t)=x(t)⊗h(t)+n(t),

where y(t) is received signal; x(t) is the modulated signal sent by the transmitter; h(t) denotes underwater acoustic channel; “⊗” denotes convolution operation; n(t) is additive noise. The impulse response function model for the multipath channel can be expressed as [27]:

(2) $$h(t)=A\delta (t-\tau_{0})+\sum_{i=1}^{N}A_{i}\delta (t-\tau_{i}),$$

where A and $A_{i}$ are amplitudes; $\tau_{0}$ and $\tau_{i}$ represent time delays. The first term on the right side of the equal sign is the direct sound wave, and the second term is bounded refraction and reflection waves.

In underwater communication systems, common modulation types include MFSK [28], multiple-phase shift keying (MPSK) [28], orthogonal frequency division multiplexing (OFDM) [28], linear frequency modulation (LFM) [28], and hyperbolic frequency modulation (HFM) [29].

## 3. System Model and Proposed Method

**Figure 1:**
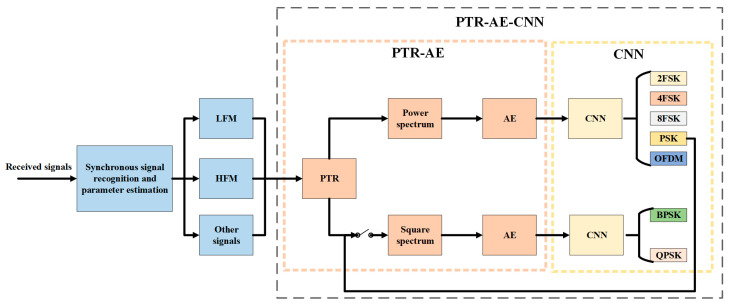
Flow chart of modulation recognition based on passive time reversal-autoencoder with synchronous signals.

The method proposed in this paper is divided into three steps: synchronous signal recognition and parameter estimation, frequency domain feature enhancement based on PTR-AE, and classification recognition. The specific process is shown in Figure 1.

First, a synchronous signal recognition network is designed to identify the type of synchronous signal. And then its parameters are estimated based on fractional Fourier transform (FrFT), Hough transform, and spectral features. Afterward, PTR-AE and CNN are designed for power spectrum features enhancement and modulation recognition, to classify the signals into 2FSK, 4FSK, 8FSK, PSK, and OFDM.

Due to the similarity of power spectrum features between BPSK and QPSK, the square spectrum is selected as the classification feature. Therefore, after identifying the modulation types of the signal as PSK, PTR-AE, and CNN are used for square spectrum features enhancement and modulation recognition of BPSK and QPSK.

## 4. Synchronous Signal Recognition

### 4.1. Structure of Synchronous Signal Recognition Network

**Figure 2:**
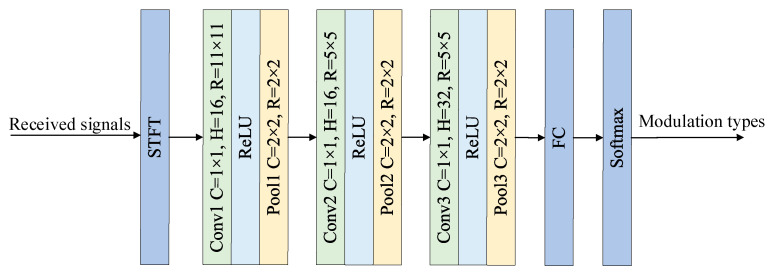
Structure diagram of synchronous signal recognition network.

Due to the significant differences in time–frequency characteristics among HFM, LFM, and other communication signals, we use the time–frequency features calculated by short-time Fourier transform (STFT) to recognize them. The specific structure and parameters of the synchronous signal recognition network are shown in Figure 2, where Conv represents the convolutional layer, C represents the size of the convolutional kernel and pooling kernel sliding step, H denotes the number of convolutional kernels, R is the convolutional kernel size and maximum pooling window size, and FC represents the fully connected layer. The synchronous signal recognition network includes the convolution layers, pooling layers, a full connection layer, and a Softmax layer. ReLU and the cross-entropy function are used as the activation function and loss function, respectively.

### 4.2. Training and Testing of Synchronous Signal Recognition Network Models

During the training process, the sampling rate is set to 96 kHz and the number of sampling points for each signal segment is 8192. Other parameters are shown in Table 1. “/” means that the parameter is not involved, “[]” indicates that the data is randomly selected within the closed set range, “(Hough 1962)” indicates random selection among the listed items, and “∪” is the union operator. The underwater acoustic channel data are generated by Bellhop, and the specific parameters are shown in Table 2.

During the training phase, 500 samples are generated for each modulation signal based on the parameters in Table 1. Bellhop is used to generate simulated underwater acoustic channels according to the parameters in Table 2. The signal-to-noise ratio (SNR) is set within the range from 0 to 10 dB.

During the testing phase, the underwater acoustic channels of Haihe and Danjiangkou reservoir are used as testing environments, with specific parameters shown in Table 3. The number of test samples for each modulation signal corresponding to each channel is 100.

**Figure 3:**
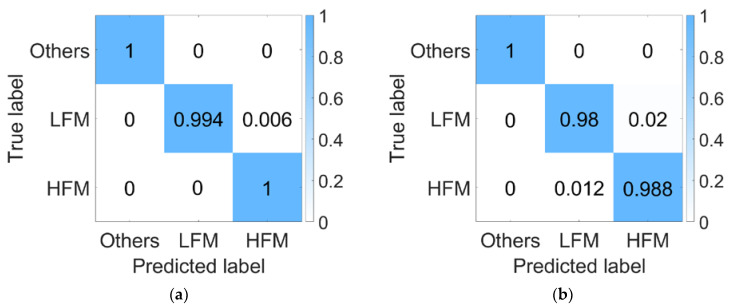
LFM and HFM signal recognition confusion matrix. (**a**) Haihe; (**b**) Danjiangkou reservoir.

Figure 3 shows the recognition accuracy of the synchronous signal recognition network in two environments. Under two different channels from the training environments, the recognition accuracy can reach over 98%.

## 5. Estimation of Synchronous Signal Parameters

### 5.1. Estimation of LFM Parameters

Fractional Fourier transform (FrFT) [30] has a good energy aggregation effect on a given LFM signal in a certain order of the fractional Fourier domain. The relationship between LFM frequency modulated rate $k_{0}$ and optimal order $p_{0}$ is:

(3) $$k_{0}=\frac{-f_{s}^{2}{}^{}}{L\tan(p_{0}\pi /2)},$$

where L is the length of the discrete signal; $f_{s}$ is the sampling rate; $p_{0}$ represents the optimal FrFT order, ranging from 0 to 4. The initial interval of $p_{0}$ can be determined based on the value of the frequency modulation $k_{0}$ displayed in the spectrogram. If $k_{0}$ is positive, the initial value range is [0, 2], and if $k_{0}$ is negative, the initial range is [2, 4). Set the search step Δp=0.01, calculate the fractional order spectrum, and obtain the rough value of $p_{0}$ based on the corresponding point of the maximum absolute amplitude. Then, within the range $\left[p_{0}-\Delta p,\;p_{0}+\Delta p\right]$, set the step size Δp=0.001 for accurate estimation. Finally, calculate $k_{0}$ according to Formula (3).

As for estimating $N_{0}$ and L, we calculate the frequency spectrum through Fourier transform first, and then set two thresholds to determine the maximum frequency $f_{\max}$ and minimum frequency $f_{\min}$ of the LFM signal. Threshold 1 and threshold 2 can be calculated by:

(4) $$X(\omega )=\sum_{n=0}^{N-1}x(n)\exp(-i\frac{2\pi \omega n}{N}),$$

(5) $${\mathrm{threshold}}_{1}=\frac{a_{1}}{N}\sum_{\omega =1}^{N}\left|X(\omega )\right|,$$

(6) $${\mathrm{threshold}}_{2}=\frac{a_{2}}{N}\sum_{\omega =1}^{N}\left|X(\omega )\right|,$$

where N is the number of sampled points of the received signal; $a_{1}$ and $a_{2}$ are threshold parameters, ranging from 0 to $\frac{\max(\left|X(\omega )\right|)}{\bar{\left|X(\omega )\right|}}$, where $\bar{\left|X(\omega )\right|}$ is the mean of $\left|X(\omega )\right|$. Let $f_{\min}$ be the first frequency point where the power is greater than threshold 1, and $f_{\max}$ be the last frequency point where the power is greater than threshold 2. The periods T and L can be calculated by:

(7) $$T=\frac{f_{\max}-f_{\min}}{k_{0}},$$

(8) L=T×fs,

Using Δa=0.1 as the step size, all $a_{1}$ and $a_{2}$ are transversed to estimate T and $f_{\min}$. The LFM signal is recovered and a cross-correlation with the received signal is performed. The $a_{1}$, $a_{2}$ and corresponding $f_{\min}$ and T are found, achieving achieve the maximum cross-correlation peak, then this correlation peak is used to determine the starting position $N_{0}$.

We test the estimation accuracy by using the channels of the Haihe and Danjiangkou reservoir. The true and estimated values are summarized in Table 4. $N_{0}$ is based on 0, with delays greater than zero and advances less than zero. When the sampling rate is 96 kHz, the estimation errors of $N_{0}$ and L do not exceed 60 sampling points, and the error percentage of $k_{0}$ is less than 1.2%.

### 5.2. Estimation of HFM Parameters

The Hough transform [31] is commonly used to detect curves in images. By using the transformation between two coordinate spaces, curves with the same shape in the coordinate space form peaks that map to points in another space. This paper estimates $k_{0}$ of HFM based on the Hough transform and time–frequency image calculated by Wigner-Ville distribution (WVD), because WVD has good energy aggregation and high resolution, it can better characterize the time–frequency characteristics of HFM.

The frequency of the HFM signal at each moment is:

(9) $$f=\frac{1}{k_{0}t+\frac{1}{f_{0}}}.$$

Convert the equation into a polar coordinate system [32]:

(10) $$\rho =t\cos\theta +\frac{\sin\theta }{f}.$$

Find the peak point $\left(\rho_{0},\theta_{0}\right)$ in the ρ−θ parameter space and calculate $k_{0}$ by [32]:

(11) $$k_{0}=-\frac{1}{\tan\theta_{0}}.$$

After obtaining the estimated value $k_{0}$, $N_{0}$ and L are estimated by using the same method as LFM. The final results are summarized in Table 5. When the sampling rate is 96 kHz, the estimation errors of $N_{0}$ and L do not exceed 90 points. The percentage error of $k_{0}$ is less than 5%.

## 6. Signal Frequency Domain Feature Enhancement Network Based on PTR-AE

To actively alleviate the impact of multipath effects on communication signal modulation recognition, PTR-AE is designed to suppress multipath effects and enhance signal frequency domain features after identifying and estimating specific parameters of the synchronous signal.

### 6.1. Passive Time Reversal Detection Signal Selection

The implementation of PTR requires two parts: detection signal and modulation signal. The detection signal needs to meet the following conditions [33]:

(1) Its frequency band must cover all frequency bands of the effective signal data;
(2) It must have good autocorrelation characteristics;
(3) Its frequency spectrum should be whitened as much as possible within the frequency band.

In the process of underwater acoustic communication, it is necessary to add a synchronization signal to assist in the synchronization and demodulation of modulated signals. Common synchronization signals include LFM and HFM, both of which meet the above conditions and can be used as detection signals for passive time reversal.

### 6.2. The Principle of Passive Time Reversal

**Figure 4:**
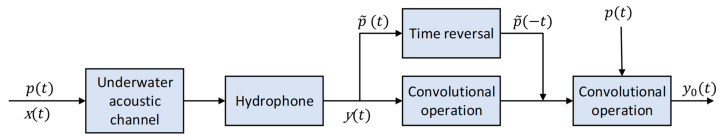
Structure diagram of synchronous signal recognition network.

The detection signal $\tilde{p}(t)$ at the receiving end is first time reversed, and then convolved with the received modulated signal y(t) to obtain intermediate data. The intermediate data is convolved with the detection signal p(t) at the sending end to suppress multipath effects. The schematic diagram is shown in Figure 4.

### 6.3. Feature Spectrum Estimation

After using synchronous signal-based PTR to suppress multipath effects on the signal, appropriate feature spectra should be selected as the input of AE. Due to the significant differences between 2FSK, 4FSK, 8FSK, PSK, and OFDM, the power spectrum is first selected as the classification feature, and AE is used to enhance it. The power spectrum P(ω) can be calculated by:

(12) $$Y(\omega )=\sum_{n=0}^{N-1}y_{0}(n)\exp(-i\frac{2\pi \omega n}{N}),$$

(13) $$P(\omega )=\frac{{\left|Y(\omega )\right|}^{2}}{N},$$

where $y_{0}(n)$ is the discrete modulated signal after passive time reversal, and N is the number of signal sampling points.

The square spectrum of the BPSK signal has an impulse characteristic at the position that is twice the carrier frequency, the QPSK signal does not have this feature. Therefore, for these two types of signals, we select the square spectrum as the modulation classification feature. AE is also used to enhance the square spectral features of these two signals. The square spectrum can be expressed as:

(14) $$\;S(\omega )=\left|\sum_{n=0}^{N-1}y_{0}{\left(n\right)}^{2}\exp(-i\frac{2\pi \omega n}{N})\right|\;.$$

### 6.4. Structure of PTR-AE

**Figure 5:**
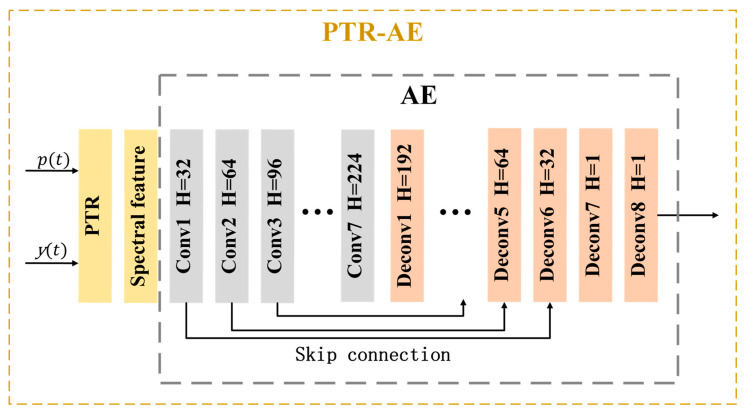
Structure of PTR-AE.

The PTR-AE consists of two parts: a passive time reversal layer and an autoencoder which consists of seven convolutional layers and eight deconvolution layers. There are some skip connections between convolutional layers and deconvolution layers. The convolutional kernel size is 15, and its sliding step size is 2. Leaky ReLU is used as an activation function. The network structure is shown in Figure 5, in which Conv represents the convolutional layer, Deconv represents the deconvolution layer, and H represents the number of convolutional kernels.

The convolutional layers of the encoder compress the input signal features layer by layer, remove redundant information, and extract high-dimensional features. The deconvolution layers of the decoder realize signal feature decoding and reconstruction. The L1 loss term is used to measure the feature enhancement effect, and the RMSProp optimizer is selected to optimize and adjust the network parameters.

## 7. CNN-Based Modulation Classification Network

**Figure 6:**
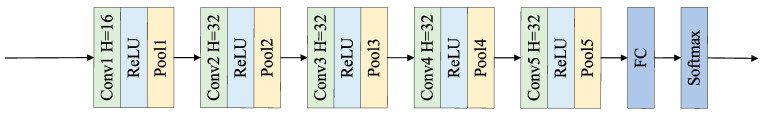
Structure of CNN.

After feature enhancement, CNN is designed for modulation classification. The network includes five convolution layers, five pooling layers, and one full connection layer. ReLU is selected as the activation function. The cross-entropy is selected as the loss function. CNN extracts high-dimensional features of the signal power spectrum and square spectrum through convolution and finally classifies them using a Softmax layer. The network structure is shown in Figure 6, where Conv represents the convolutional layer, Pool represents the pooling layer, and H represents the number of convolutional kernels. The convolutional kernel size is five, and the sliding step size is one. The maximum pooling size is two.

By learning the enhanced signal power spectrum features, CNN can classify 2FSK, 4FSK, 8FSK, PSK, and OFDM. When the modulation type of the signal has been identified as PSK, the enhanced square spectral features and CNN are used to further classify BPSK and QPSK.

## 8. Training and Testing of PTR-AE-CNN

### 8.1. Training of PTR-AE-CNN

During the training process, the sampling rate is set to 96 kHz. Except for OFDM and detection signals, the duration of all other signals is 20 ms, and the duration of detection signals LFM and HFM is 50 ms. Other parameters are shown in Table 6, where “/” indicates that the parameter is not involved, “[]” indicates that the data is randomly selected within the closed set range, and “{}” indicates that it is randomly selected among the listed items. The underwater acoustic channel is generated by Bellhop, and the specific parameters are shown in Channel 3 in Table 2.

In total, 500 sending samples are generated for each modulation signal, and 600 channels are generated by Bellhop. Received data are generated according to Formula (1) with SNR set from 0 to 10 dB. The network parameters of PTR-AE and CNN are constantly optimized through training data, and the training is stopped when the loss function becomes stable.

### 8.2. Performance Testing of PTR-AE-CNN

During the testing phase, three types of underwater acoustic channels, Haihe, Danjiangkou reservoir, and BCH1 channel, data provided by Watermark [34], are used as testing environments. The corresponding number of test samples for each modulation signal in both environments is 600. The specific parameters of the test channel are shown in Table 3.

**Figure 7:**
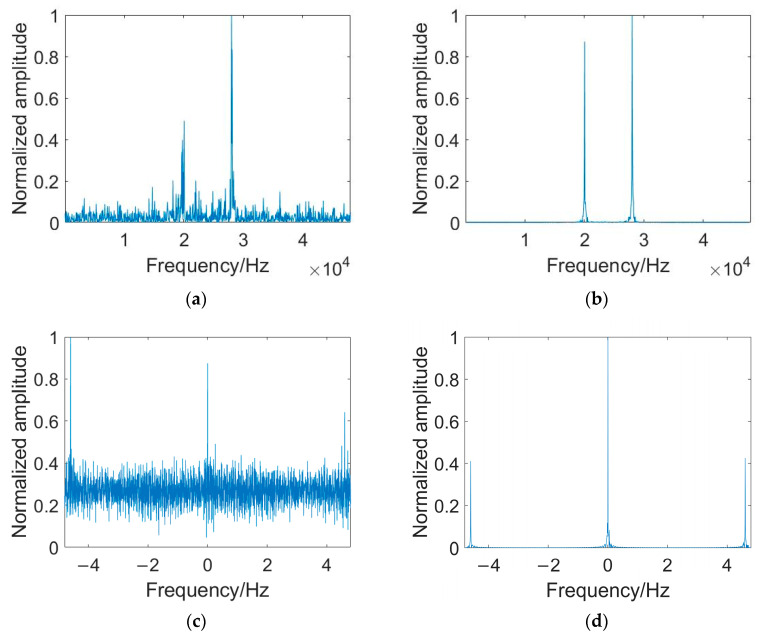
Enhancement effect of PTR-AE on frequency-domain characteristics of modulated signals when the detection signal is LFM. (**a**) Power spectrum of 2FSK received signal; (**b**) Power spectrum of 2FSK signal enhanced by PTR-AE. (**c**) The square spectrum of BPSK received signal; (**d**) The square spectrum of BPSK signal enhanced by PTR-AE.

**Figure 8:**
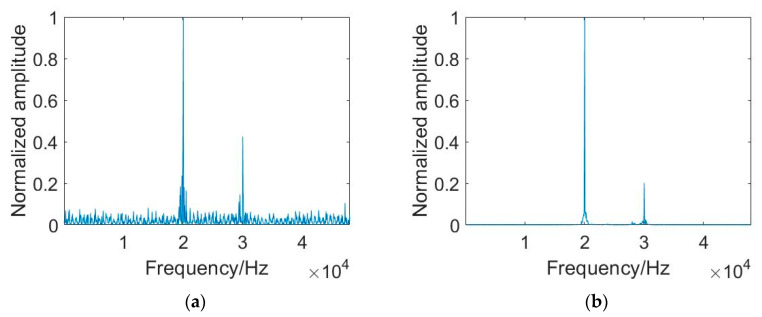

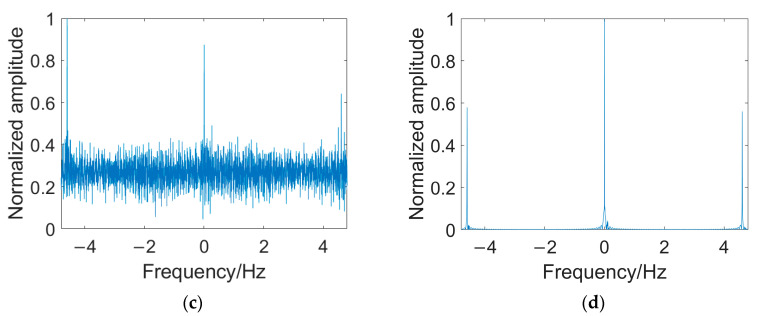
Enhancement effect of PTR-AE on frequency domain characteristics of modulated signals when the detection signal is HFM. (**a**) Power spectrum of 2FSK received signal; (**b**) Power spectrum of 2FSK signal enhanced by PTR-AE. (**c**) The square spectrum of BPSK received signal; (**d**) The square spectrum of BPSK signal enhanced by PTR-AE.

Taking 2FSK and BPSK signals as examples, the enhancement effect of PTR-AE on signal frequency-domain features in this paper is shown in Figure 7 and Figure 8. After PTR-AE enhancement, the spectral line characteristics of the 2FSK signal’s power spectrum in the frequency range of 20–30 kHz are clearer. The impulse characteristics of the BPSK signal at the square spectrum double carrier frequency position are enhanced. When the detection signal is LFM, the average SNR of the power spectrums of the six signals increases from 1 dB to 7 dB, and the average SNR of the PSK square spectrums increases from 1.5 dB to 11 dB. When the detection signal is HFM, the average SNR of the signal power spectrums increases by 6.14 dB, and the average SNR of the square spectrums increases by 10.5 dB.

**Figure 9:**
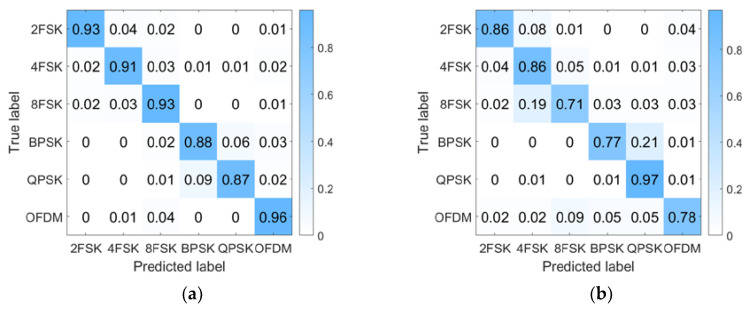

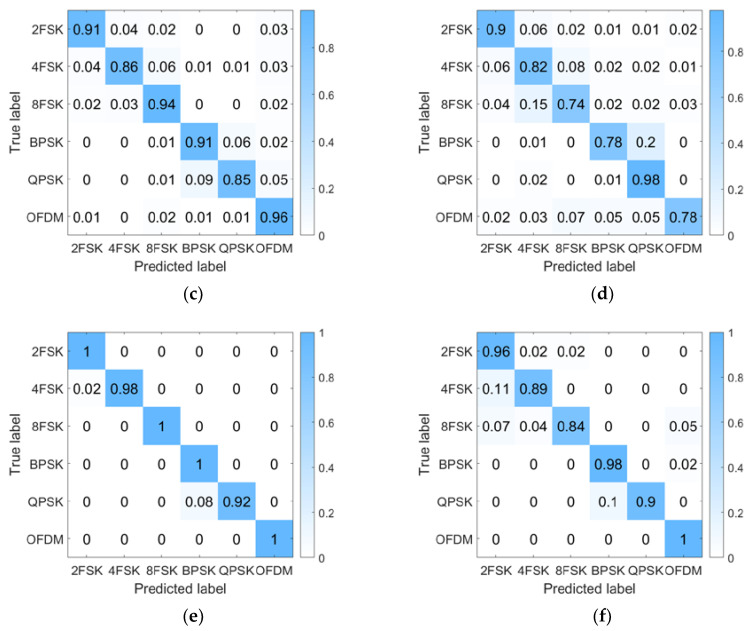
Identification confusion matrix of six modulation signals. (**a**) Haihe (LFM is the detection signal); (**b**) Haihe (HFM is the detection signal); (**c**) Danjiangkou reservoir (LFM is the detection signal); (**d**) Danjiangkou reservoir (HFM is the detection signal); (**e**) BCH1 (LFM is the detection signal); (**f**) BCH1 (HFM is the detection signal).

Figure 9 shows the recognition accuracy of the proposed method in three different environments. When the detection signal is LFM, in the Haihe River, the OFDM signal recognition accuracy is greater than 80%, and the accuracy of other modulation signals is higher than 90%. In the Danjiangkou reservoir, the accuracy of all modulated signals is higher than 85%. When using BCH1 channel data testing, the recognition accuracy of all signals is above 90%.

When the detection signal is HFM, the accuracy is slightly lower than LFM. This is due to the energy distribution of the HFM spectrum not being as uniform as LFM, which affects the passive time reversal to some extent. But overall, the recognition rate of all signals is above 70%, and it is also adaptable to changes in underwater acoustic channels.

To demonstrate the effectiveness of PTR-AE in improving the accuracy of modulation recognition, we compared the effectiveness of using CNN for modulation recognition without using PTR-AE for feature enhancement processing under the environment of Haihe and Danjiangkou reservoir. It can be seen from the results that the modulation recognition accuracy is improved by at least 20% after PTR-AE enhancement. We also compare it with methods based on Resnet [16], DAE-Alexnet [21], Alexnet [35], and R&CNN [15]. Table 7 summarizes the test results and parameter quantities of these methods.

The above results indicate that the method in this paper can effectively suppress the impact of multipath effects, significantly enhance the frequency-domain characteristics, and the model is robust to changes in environmental conditions. Traditional passive modulation recognition methods lack effective signal enhancement processing, and the input data of the classifier is severely disturbed by the underwater acoustic channel, which results in fuzzy features. The recognition accuracies of these models are very sensitive to the changes in underwater channels and require training with a small amount of data from the testing environment to achieve the desired effect. In this paper, we actively utilize synchronous signals to suppress multi-path effects, improve the input of traditional classifiers, reduce the model’s dependence on environmental conditions, and achieve good recognition accuracy without adjusting model parameters. Since both PTR-AE and CNN use one-dimensional convolutional kernels, only the synchronous signal recognition network uses a two-dimensional convolutional kernel, compared with other comparison methods in this article, the number of parameters in neural networks is not very large.

### 8.3. Testing the Impact of Signal Synchronization and Length Error on the Model

This section tests the impact of the estimation errors of $N_{0}$ and L on the model recognition accuracy. The range of $N_{0}$ is [−100, 100], where less than 0 indicates an early synchronization position, greater than 0 indicates a delayed synchronization position, the range of L values is [4700, 4900], and the sampling rate is set to 96 kHz.

**Figure 10:**
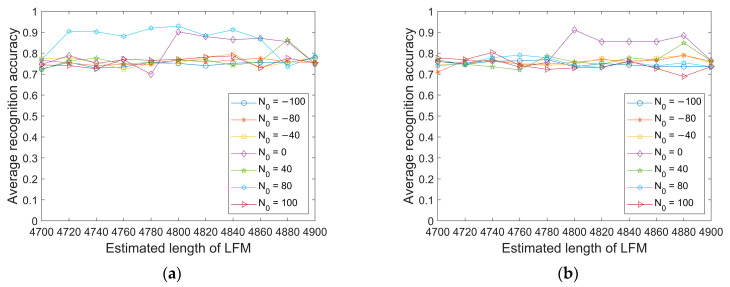
The average recognition accuracy of six modulation signals under two test environments with errors in LFM parameter estimation. (**a**) Danjiangkou reservoir; (**b**) Haihe.

**Figure 11:**
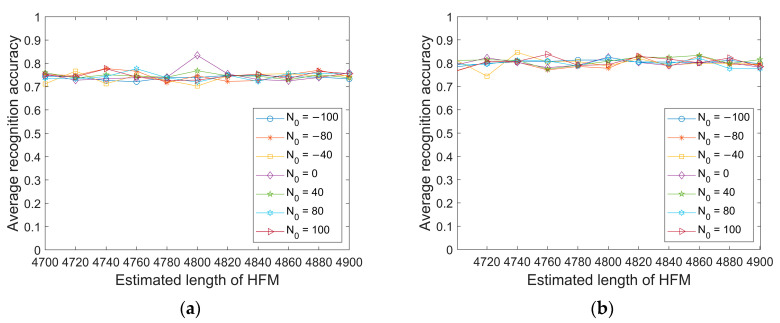
The average recognition accuracy of six modulation signals under two test environments with errors in HFM parameter estimation. (**a**) Danjiangkou reservoir; (**b**) Haihe.

From Figure 10 and Figure 11, it can be seen that under the condition of a sampling rate of 96 kHz, when the position and length errors of the synchronous signal are controlled within the range of 100 sampling points, the average recognition accuracy of the six modulation signals decreases to a limited extent, and the overall average recognition rate is still higher than existing passive modulation recognition methods.

## 9. Discussion

### 9.1. Significance of the Proposed Method

The method proposed in this article provides a new approach to improve the accuracy of modulation recognition. Actively utilizing synchronous signals commonly used in underwater acoustic communication as detection signals, a passive time reversal autoencoder is designed to enhance signal features, improving the accuracy of modulation recognition and environmental adaptability.

The proposed model can also be used for modulation recognition of other types of signals, but suitable features need to be selected based on specific signals to better leverage the advantages of the model itself.

### 9.2. Future Research Direction

In the estimation of synchronous signal parameters, the method of estimating frequency modulation parameters based on FrFT and Hough transform is relatively mature. However, the method used in this paper is relatively simple for estimating signal starting position and length. In addition, the effect of passive time reversal mirrors is affected by noise. Under low SNR conditions, it is necessary to adopt noise suppression preprocessing to ensure the feature enhancement effect of PTR-AE.

In the future, more detailed research can be conducted on the parameter estimation problem of synchronous signals to further reduce parameter estimation errors. At the same time, effective denoising methods should also be studied to improve the enhancement effect of PTR-AE on signal features under low SNR conditions.

## 10. Conclusions

To reduce the impact of multipath effects on the accuracy of modulation recognition in the ocean, this paper actively utilizes synchronous signals in underwater acoustic communication to suppress the multipath effect. A passive time reversal-autoencoder based on synchronous signals is designed to enhance signal characteristics in underwater acoustic channels. Modulation classification is performed using the convolutional neural network.

The results show that PTR-AE can suppress multipath effects in underwater acoustic channels, and enhance power spectrum and squared spectrum features. It can also show good recognition performance in different underwater acoustic channels. Compared with existing methods, the modulation recognition rate of this article has been improved by at least 10%.

## Figures and Tables

**Table 1:** Modulation signal parameters.

Modulation Types	Symbol Rate/(Symbol·s^−1^)	Carrier Frequency/kHz	Number of Subcarriers	Frequency Modulated Ratio/(kHz·s^−1^)	Bandwidth/kHz
2FSK	{800, 1000}	[20, 30]	/	/	/
4FSK	{400, 500}	[20, 30]	/	/	/
8FSK	{400, 500}	[20, 30]	/	/	/
BPSK	1000	25	/	/	/
QPSK	1000	25	/	/	/
OFDM	/	[20, 30]	1024	/	10
LFM	/	[20, 30]	/	[100,000, 200,000]∪[−200,000, −100,000]	[8, 10]
HFM	/	[20, 30]	/	[1/6000, 1/2800]∪[−1/2800, −1/6000]	[8, 10]

**Table 2:** Underwater acoustic channel parameters during the training phase of synchronous signal recognition network.

Parameters	Channel 1	Channel 2	Channel 3
Depth/m	100	100	100
Sending height/m	50	20	50
Receiving height/m	60	50	60
Distance/m	1000	1000	1300

**Table 3:** Underwater acoustic channel parameters in the test phase.

Parameters	Haihe	Danjiangkou Reservoir
Depth/m	8	53
Sending height/m	6.5	43
Receiving height/m	6.5	43
Distance/km	1	0.5

**Table 4:** Estimated values of LFM parameters in the channels of Haihe and Danjiangkou reservoir.

Parameters	Sending Signals	Haihe	Danjiangkou Reservoir
$N_{0}$	0	17	52
L	4800	4780	4849
$f_{\min}$	20	19.93	20.12
$f_{\max}$	30	29.933	30.11
$k_{0}$/(kHz·s^−1^)	200	200.9	197.79
$p_{0}$	1.066	1.065	1.064

**Table 5:** Estimated values of HFM parameters in the environment of Haihe and Danjiangkou reservoir.

Parameters	Sending Signals	Haihe	Danjiangkou Reservoir
$N_{0}$	0	−82	−43
L	4800	4711	4864
$f_{\min}$/kHz	20	19.795	19.718
$f_{\max}$/kHz	30	29.952	30.278
$k_{0}$/(Hz·ms^−1^)	−0.33333	−0.34907	−0.34907
*ρ*	4.1667 × 10^−5^	4.2274 × 10^−5^	4.2274 × 10^−5^
θ/°	89.9809	89.98	89.98

**Table 6:** Modulation signal parameters.

Modulation Types	Symbol Rate/(Symbol·s^−1^)	Carrier Frequency/kHz	Number of Subcarriers	Frequency Modulated Rate/(kHz·s^−1^)
2FSK	{800, 1000}	[20, 30]	/	/
4FSK	{400, 500}	[20, 30]	/	/
8FSK	{400, 500}	[20, 30]	/	/
BPSK	1000	25	/	/
QPSK	1000	25	/	/
OFDM	/	[20, 30]	1024	/
LFM	/	[20, 30]	/	2 × 10^5^
HFM	/	[20, 30]	/	−3.3333 × 10^−4^

**Table 7:** The modulation recognition accuracy and parameters quantities of different methods.

Methods	Haihe	Danjiangkou Reservoir	Parameter Quantities
PTR-AE-CNN(LFM)	91.1%	90.2%	6,968,000
PTR-AE-CNN(HFM)	82.6%	83.4%	
CNN(LFM)	50.4%	39.9%	49,000
CNN(HFM)	57.1%	60.0%	
Resnet [16]	17.7%	18.5%	53,000
DAE-Alexnet [21]	68.2%	70.2%	118,450,000
Alexnet [35]	70.2%	48.2%	61,100,000
R&CNN [15]	16.7%	16.6%	7,672,000

## Data Availability

The data presented in this paper are available after contacting the corresponding author.
